# A qualitative study of microaggressions against African Americans on predominantly White campuses

**DOI:** 10.1186/s40359-020-00472-8

**Published:** 2020-10-23

**Authors:** Monnica T. Williams, Matthew D. Skinta, Jonathan W. Kanter, Renée Martin-Willett, Judy Mier-Chairez, Marlena Debreaux, Daniel C. Rosen

**Affiliations:** 1 School of Psychology, University of Ottawa, 136 Jean-Jacques Lussier, Vanier Hall, Ottawa, Ontario K1N 6N5 Canada; 2 Department of Psychology, Roosevelt University, Chicago, USA; 3 Department of Psychology, University of Washington, Seattle, USA; 4 Department of Psychology and Neuroscience, Institute of Cognitive Science, University of Colorado Boulder, Boulder, USA; 5 Department of Psychology, University of Louisville, Louisville, USA; 6 Department of Human and Organizational Development, Vanderbilt University, Nashville, USA; 7 Department of Counseling & Health Psychology, Bastyr University, Kenmore, USA

**Keywords:** Microaggressions, Discrimination, Taxonomy, Ethnic minorities, African Americans, Racism

## Abstract

**Background:**

Pierce’s (The Black seventies: an extending horizon book, 1970) conception of “subtle and stunning” daily racial offenses, or microaggressions, remains salient even 50 years after it was introduced. Microaggressions were defined further by Sue and colleagues (Am Psychol 62:271, 2007), and this construct has found growing utility as the deleterious effects of microaggressions on the health of people of color continues to mount. Microaggressions are common on campuses and contribute to negative social, academic, and mental health outcomes.

**Method:**

This paper explores how Black college students’ experiences correspond to or differ from the microaggression types originally proposed by Sue et al. (Am Psychol 62:271, 2007). Themes were identified from focus group data of students of color (*N* = 36) from predominately White institutions (PWIs) of higher learning (*N* = 3) using interpretative phenomenological analysis.

**Results:**

We identified 15 categories of racial microaggressions, largely consistent with the original taxonomy of Sue et al. but expanded in several notable ways. New categories in our data and observed by other researchers, included categories termed Connecting via Stereotypes, Exoticization and Eroticization, and Avoidance and Distancing. Lesser studied categories identified included Sue et al.’s Denial of Individual Racism, and new categories termed Reverse Racism Hostility, Connecting via Stereotypes, and Environmental Attacks.

**Discussion:**

While previous literature has either embraced the taxonomy developed by Sue and colleagues or proposed a novel taxonomy, this study synthesized the Sue framework in concert with our own focus group findings and the contributions of other researchers. Improving our understanding of microaggressions as they impact people of color may better allow for improved understanding and measurement of this important construct.

## Background

Almost 50 years ago, Pierce [33] sought to unpack the mechanism of “subtle and stunning” daily racial offenses, known as microaggressions. Pierce’s seminal description of the construct of microaggressions laid the groundwork for a re-framing by Sue et al. [41], who defined racial microaggressions as subtle, daily, and unintentional racial slights committed against people of color because they are members of a racialized group. Sue et al. [41] proposed nine categories of racial microaggressions, described as (a) assumptions that a person of color is not a true American; (b) assumptions of lesser intelligence; (c) statements that convey colorblindness or denial of the importance of race; (d) assumptions of criminality or dangerousness; (e) denials of individual racism; (f) promotion of the myth of meritocracy; (g) assumptions that one’s cultural background and communication styles are pathological; (h) being treated as a second-class citizen; and (i) having to endure environmental messages of being unwelcome or devalued. Since Sue and colleagues’ taxonomy was proposed, numerous researchers have examined these categorizations, finding generally similar but not identical groupings based on qualitative and factor analytic studies (e.g., [28, 44]).

There has been a surge of qualitative, quantitative, and theoretical work (reviewed by [53]) expanding our understanding of the nature, experience and consequences of microaggressions since Sue and colleagues proposed their taxonomy of microaggressions in 2007 [41]. Microaggressions towards targeted racial or ethnic groups are persistent and occur frequently in academic settings [40]. They are increasingly recognized to compound with the intersections of gender, sexual orientation, and other stigmatized identities (e.g., [2, 22, 46]), and these additional identities have been incorporated into Sue et al’s. original taxonomy [42]. As of this writing, multiple overlapping yet distinct taxonomies have been proposed, including several with empirical support (e.g., [18, 24, 28, 44]). In this paper, we examine the experience of racial microaggressions among African American college students and provide a qualitative description of the resulting taxonomy using focus group data, comparing our findings to Sue and colleagues [41] original work and the expanded set of themes in Sue and Spanierman [42].

### Nature and maintenance of microaggressions

Although there has been some debate about the nature of microaggressions and how they should be defined, we contend that microaggressions are actual things that can be identified and measured, and not simply the subjective experience of the target (i.e., [47]), although this not universally agreed upon by scholars. Due to the subtle nature of microaggressions, they are sometimes minimized as simple cultural missteps or racial faux pas [47]. Microaggressions are not, however, innocuous gaffes but are a form of oppression that reinforces existing power differentials between groups, whether or not this was the conscious intention of the offender [35, 47]. This reinforcement of a power differential contributes to the maintenance of microaggressions because it favors the in-group and, in an effort to retain the extant power structure, outgroup members are punished socially when they challenge microaggressions. Essed [11] has written extensively about “everyday racism,” as a tripartite framework whereby racist practices involve the marginalization of those identified as racially or ethnically different; the problematization of those cultures and identities; and repression of (potential) resistance against racism through humiliation or aggression. Simply put, racial microaggressions are a subtle and common form of racism that maintains White supremacy.

### Harms of microaggressions

The cumulative “day-to-day stress” caused by microaggressions [33] has been reliably associated with negative physical and emotional health outcomes for decades. Multiple studies indicate significant associations between experiencing microaggressions and higher levels of depression [18, 30] anxiety [51], posttraumatic stress disorder symptoms [52], impaired psychological wellbeing [1, 16], and decreased self-esteem [9, 31]. Studies also have demonstrated a relationship between discriminatory stress and physical ailments, including hypertension [8], hypothalamic-pituitary-adrenal (HPA) axis dysfunction [19], higher body mass index [21], and coronary heart disease [36].

Microaggressions are damaging to young people and adults alike, and the context of students of color in academic settings is particularly salient. School campuses in the United States have long been recognized as sites that magnify racial tensions present in the broader society [17], and young people may be exposed to racially-focused situations and conversations more frequently at school than at home [43]. Studies of college students have demonstrated a significant relationship between racial discrimination and increased substance use [4, 34], delinquency [6], decreased academic outcomes [20] and self-esteem [54]. Depression symptoms have been found to mediate a relationship between racial microaggressions and suicidality in students [32], raising grave concerns about the consequences of daily microaggression-related stress on young people.

### Focus of this study

Given the many harm of microaggressions [48], it is critical that we understand the experience of the targets of microaggressive actions. As the field moves towards the development of effective tools for the reduction of racial microaggressions, our taxonomies must be based increasingly on empirical findings for greatest utility in identifying both their occurrence and the harms that occur as a result. This paper seeks to elucidate the experience of racial microaggressions experienced by Black students at predominately White institutions of higher learning (PWIs) with a focus group design utilizing qualitative data and compare findings to Sue and colleagues’ taxonomies of racial microaggressions. The question being explored was how current Black college students’ experiences might correspond to or differ from these original and revised types.

## Method

### Recruitment

Study participants were recruited from one private and two public PWIs located in the Southern/Midwestern and Pacific Northwest United States. Participants were recruited using a combination of posted fliers, the undergraduate psychology recruiting pool, and word of mouth. Interested parties were directed toward an online screening tool, which included a demographic questionnaire. The lead research assistant contacted eligible participants to schedule a time for focus group participation. Study eligibility criteria were self-identification as Black, African American, Biracial (with Black), or Continental African.

The Institutional Review Boards’ (IRB) Social and Behavioral Sciences Committees for the various institutions approved the research, and all respondents were consented according to the rules and regulations of their respective IRBs. The informed consent procedure included a preamble consent provided online for the initial collection of demographic and self-report data, and a written form that was reviewed with researchers and signed right before the focus groups commenced.

### Analytical approach

The research team selected an interpretative phenomenological analysis (IPA) approach to codify the data [10]. This method of analysis is well suited to small homogeneous samples and the use of a semi-structured interview format. IPA utilizes “prompts and funneling” to identify and connect themes among individual perspectives on how study participants make sense of their social and personal worlds [37]. Data from each of the focus groups were audio recorded and transcribed verbatim by the research team. After the initial transcription, each transcription was checked by a second trained research assistant.

### Focus groups

The research team consisted of a diverse group of clinical psychologists, graduate assistants, and research assistants. Each of the focus groups was conducted by either one of the principal investigators or an advanced level graduate or research assistant. In every case, at least one of the facilitators was an African American faculty member.

Background information about the definition and types of microaggressions were provided during each focus group, with some examples from Sue et al.’s [41] paper. Focus group questions centered on the participants’ experience with racial microaggressions, with six initial prompts and a variety of suggested subprompts that might be used to clarify discussion following each prompt, though facilitators were encouraged to also explore topics that organically arose in the room. The primary prompts used in each group were:

- What examples of microaggressions have occurred in your life?
- What examples of microaggressions have you witnessed or heard about in friends, family or others in your life?
- Are there certain situations where you are more likely to experience a microaggression?
- What do you do?
- How do you cope?

### Participants

The final sample consisted of 36 undergraduate and graduate students from 219 students who completed the online screening survey. A total of six focus groups were conducted across three campuses, with group sizes from 2 to 11 participants. The mean age was 23.0 (SD = 6.64) with 84.4% of the participants identifying as African American, 3.1% of the participants were Latino/a, and 68.8% were female.

### Data analysis

Initial coding was completed exclusively by two pairs of graduate and undergraduate students at the institutions where the focus groups occurred. The initial coding results were reviewed and discussed by the coders as well as the faculty research team, which included an expert on racial microaggressions (MTW), as well as one with IPA experience who had trained the team (MDS). During these discussions, themes would be explored for convergence, and sent back to the coders to determine if consensus might be reached that the codes appeared to capture the meaning that each team intended. Finally, the identified themes were compared to the theoretical groupings of microaggressions from Sue et al.’s [41] taxonomy and reviewed a final time by the authors to further verify agreement across the study team on the categorization of the data. This allowed for the consideration of whether terms that already existed in the literature might serve as better labels that would bridge these results to existing literature, as well as to avoid unclearly renaming a well-described phenomenon.

## Results

The following section details the 15 resultant categories from our IPA analysis, which are also summarized in Table 1.

**Table 1:** Categories of microaggressions

	Category Name	Description
1	Not a True Citizen	When a question, statement, or behavior indicates that a person of color is not a real citizen or a meaningful part of our society because they are not White.
2	Racial Categorization & Sameness	When a person is compelled to disclose their racial group to enable others to attach pathological racial stereotypes to the person; includes the assumption that all people from a particular group are alike.
3	Assumptions About Intelligence, Competence, or Status	When behavior or statements are based on assumptions about a person’s intelligence, competence, education, income, social status derived from racial stereotypes.
4	False Colorblindness / Invalidating Racial or Ethnic Identity	Expressing that individual’s racial or ethnic identity should not be acknowledged, which can be invalidating for people who are proud of their identity or who have suffered because of it.
5	Criminality or Dangerousness	Demonstrating belief in stereotypes that people of color are dangerous, untrustworthy, likely to commit crimes or cause bodily harm.
6	Denial of Individual Racism	When a person tries to make a case that they are not biased, often by talking about anti-racist things they have done to deflect perceived scrutiny of their own behaviors.
7	Myth of Meritocracy / Race is Irrelevant for Success	When someone makes statements about success being rooted in personal efforts and denial of existence of racism or White privilege.
8	Reverse Racism Hostility	Expressions of jealousy or hostility surrounding the notion that people of color get unfair advantages and benefits due to their race.
9	Pathologizing Minority Culture or Appearance	When people criticize others based on perceived or real cultural differences in appearance, traditions, behaviors, or preferences.
10	Second Class Citizen / Ignored & Invisible	When people of color are treated with less respect, consideration, or care than is normally expected or customary. This may include being ignored or being unseen/invisible.
11	Connecting via Stereotypes	When a person tries to communicate or connect with a person through use of stereotyped speech or behavior, to be accepted or understood. Can include racist jokes and epitaphs as terms of endearment.
12	Exoticization and Eroticization	When a person of color is treated according to sexualized stereotypes or attention to differences that are characterized as exotic in some way.
13	Avoidance and Distancing	When people of color are avoided or measures are taken to prevent physical contact or close proximity.
14	Environmental Exclusion	When someone’s racial identity is minimized or made insignificant through the exclusion of decorations, literature, or depictions of people that represent their racial group.
15	Environmental Attacks	When decorations pose a known affront or insult to a person’s cultural group, history, or heritage.

### Not a true citizen

This type of microaggression, first described by Sue et al. [41] as “Alien in Own Land,” is based on assumptions that the target is not a “true American,” reinforcing notions that non-Whites are probably immigrants. Communicating exclusion, illegitimacy, and lack of belonging that can make people of color feel like outsiders. This type of microaggression has often been described in relation to the experiences of Asian and Latino/a Americans, but African American focus group participants reported experiencing it as well. Some exemplars of this theme are as follows:

*My head is shaved, actually. I hear a lot of things. It’s really annoying about… a lot of people, especially from my friends. They think that I shaved my head for like, “Oh, do shave your head for a religious thing? Or like an African ritual or something?” Because they see, like you know, like the pictures and stuff in the media. And they automatically assume that like, and I hear it a lot too… “Oh, where are you from? Are you from like Africa?” – Female respondent*

*Just a few months ago, someone who works in the office of admissions was with students from Tanzania and she asked me where I was from. I said Cadence, Kentucky, and she said “No, before that, where are you from?” And I said, “Kentucky. I was born here. And she said, ‘No seriously your name is [redacted] so you have to be from outside of America. But we were in the presence of Tanzanian students who obviously didn’t want to be asked where they were from.” – Female respondent*

### Racial categorization & sameness

This category is intended to describe the microaggressions that occur when a person of color is compelled to disclose their racial identity to others, often leading to the expression of pathological stereotypes based on that identity (e.g., the next category; [49]). It also applies when people make comments or assumptions that people of a given race are all alike. This results in the harmful ascription of stereotypes that may serve to disconnect an individual from their actual heritage or lived experience, to incorrectly ascribe attributes to one’s heritage or experience, or to force unwanted attributes or group responsibility to an individual.

*I was folding t-shirts one day and this boy came up to me and said, [deepens voice] “I’ve been meaning to ask you this, what are you?” I said, “I’m a girl.” He was like, “No, no, no, no, no. What’s your race?” I’m like, “I’m Black.” He was like, “No way, no way!” I’m like, “If you see my entire family, I’m literally Black.” And I don’t think I look anything but Black, but he like wouldn’t believe it. – Female respondent*

*Sometimes my friends are like, “You’re so White,” or something like that. And I mean, I’m half-White. But I’m just like annoyed with it. I know I’m mixed, I understand that I am Black and White. I don’t have to act a certain way. – Female respondent*

*…I always find that whenever a topic of fashion comes up, people are like, “Why do Black people droop their pants?” And it’s just like I speak for all Black people because I’m Black? Maybe you should ask someone who is drooping their pants? I don’t know. – Female respondent*

Further, those who identify as biracial or multiracial may struggle to feel accepted, feel confused about their identity, and may experience social pressure to align with a single identity [52]. Intersectionality due to characteristics like gender, sexual identity, or religion may also be overlooked. One student shared her frustration during the group discussion.

*And they’ll bring up that we’re being divisive. But we’re like, “You need to recognize that black Muslims exist and issues about Muslim people, I guess, probably do affect us. But the issues regarding Black people, like police brutality, also affect us.” But they just seem to forget that. They just ignore it. – Female respondent*

### Assumptions about intelligence, competence, or status

This category is intended to include any positive or negative ascriptions of intellectual abilities, competence, education, or social standing based on racial assumptions. A common phenomenon encountered in the focus groups was counter-stereotypical surprise or assumed exceptionalism (e.g., “You’re not like other Black people.”) Many African American focus group respondents reported they encountered disbelief when they demonstrated academic excellence or expressed professional career ambitions.

*We were deciding where we wanted to go to college, and these people were supposed to help us. And she, like I went in there, and I was like, “Oh, here are the places I want to apply to and I’m interested in.” And she’s like, “I think you should look at community colleges.” I’m like, “What are you talking about?” I had a 3.7 GPA – what do you mean I should look at community colleges?! I was like, “Well, I’m not really interested in community college, this is my list I’ve already decided.” She was like, “No, let’s look at this,” and it was some random college I had never heard of like in the middle of nowhere. I got really upset. I went home, I cried. – Female respondent*

*On the first day of class you get a lot of looks like “Are you in the right class? Why are you here?” – Female respondent*

*People will say sometimes, “Oh, you have a Master’s.” Like they feel so surprised when they find out that you are actually educated or you have a higher degree. And when she said that to me, “Oh, you’re such a good writer.” I just didn’t know how to respond, cause I’ve heard that said to me by other people that are White, and to me it was not a compliment. I was just kind of like, “Well, did you not think that I would be a good writer? Is there a reason why I wouldn’t be a good writer?” – Female respondent*

### False colorblindness / invalidating racial or ethnic identity

Colorblindness includes statements that indicate a person does not want to acknowledge race and instead focus on shared humanity [41]. Not all forms of colorblindness were considered negative by focus group participants, who often welcomed the idea that they could be treated equally by others rather than being racialized. However, they did not typically believe that their race was unseen or unnoticed by those professing colorblindness. Thus, the category is renamed here as “false colorblindness,” to account for the various examples in the data where “colorblindness” functionally prevents honest discussions about important racial issues.

*I think just from professors as well as from students that are like, “Oh I don’t see color” or ignoring the fact that they might have said something that was bigoted or racist and not acknowledging that they have an issue or being able to address people as people, and also acknowledging that they have different ethnicities and different ethnic backgrounds. – Female respondent*

*I’ve taken a lot of women and gender studies classes, and it’s [privilege] a topic that we talk about a lot…And I don’t know if I’m the only Black person in that class, there may be two or three more, but definitely not a lot of us. And when we’re discussing it there are a lot of people who are like, “Yeah, I don’t know. I just can’t see it from y’all’s point of view.” – Female respondent*

*I told my roommate that I was going to a Black Lives Matter event, and he said, “No bro, it’s all lives matter.” And I was like, “Ah, come on man. Where are all of the All Lives Matter events? Who is doing the All Lives Matter protests?” – Male respondent*

### Criminality or dangerousness

This occurs when someone demonstrates belief in or acts on stereotypes that people of color are dangerous, untrustworthy, or likely to commit crimes or cause bodily harm (e.g., [49]). It could also include concerns about being treated badly by people of color (i.e., verbal aggressions) leading to emotional harm. This category garnered the largest number of comments from focus group participants.

*It’s like an everyday thing. Well, not necessarily every day, but just like on campus like… I remember I was walking over toward the law school to meet somebody… Like I’m walking, and this White dude was in front of me, and he looked back and seen me walking behind him and he literally kept [checks over each shoulder] looking over his shoulder like I was going to do something. – Male respondent*

*I was coming down the stairs and this really tall White guy was going up [the stairs] and I was in his way. He flinched! I’m 5’3”, I’m not going to injure you. – Female respondent*

*Another instances when I see microaggressions play out is in the differences in the way that you dress. I can dress in you know a pair of Timbs or a pair of boots and just a baggy sweatshirt. You go out because I’m feeling a little lazy that day and have everybody walk to the other side of the street. But the second I put on a button down and a little bit tighter pants and some dress shoes or you know something along the lines of that then everybody feels a little bit more comfortable, you know, and it’s like why is it that all of a sudden my perception is changing just because of the attire that I have on? – Female respondent*

### Denial of individual racism

In contrast to False Colorblindness, this type of microaggression occurs when a person tries to make a case that they do not have any racial biases, which may be triggered by perceived scrutiny of the offender’s behavior. This may take the form of talking about anti-racist things the person has done or connections the person has with other people of color. This category was supported by our data, however, and when employed as a response to criticism, it can be invalidating to people of color who are trying to draw attention to a problematic behavior (e.g., [26]).

*I was dating a [White Bulgarian]guy for a little bit. … every time we hung out he’d be like “Yeah, I have lot of Black friends. I listen to this kind of rap music.” And I’m like, “Why are you telling me this?” Like, ok, I guess I don’t know if he was trying to fix something. – Female respondent*

*When I bring it up to them, about something, they kinda say, “Well, it’s not anything offensive.” Well some of them think of themselves as being Black, like some girls I know really have this identity crisis, where they just think they can relate so much to our culture that they are like… They want to be Black, like they date the Black boys and stuff like that, so they feel like the comments they make don’t matter, because they feel like they already are within our culture. But they don’t understand like, if you understood what it meant to be us, you wouldn’t make comments like that towards us. – Female respondent*

### Myth of meritocracy / race is irrelevant for success

This microaggression occurs when people deny the ongoing existence of systemic racism or harmful discriminatory behavior, specifically in regard to personal achievement or barriers to achievement. They embrace the myth of meritocracy and the notion that the determinants of success are unequivocally rooted in personal efforts, refuting that White privilege is an unearned benefit resulting in tangible differences in outcomes at a personal or societal level.

*I’ve had a mixed girlfriend of mine sit there and say … “Black people are just blaming the system, and they just need to take advantage of, you know, the opportunities they have.” And it’s like well really what – how many opportunities do we have? Can you sit up here and put on a list of how many opportunities we as African Americans have compared to all the opportunities that Whites have, or you know, Asian Americans or Mexican Americans? Because if you sat up there and compared the list, our list is going to be pretty short. You know, can you explain to me why it is that we have [so many] African American men in prisons, a lot of African American women in prisons who still haven’t gone through trial, and it’s two years later that they’ve been sitting in jail. I know friends who have seen their friends sitting in jail awaiting trial for two years. – Female respondent*

### Reverse racism hostility

This microaggression includes expressions of jealousy or hostility surrounding the notion that people of color get unfair advantages and benefits due to their race, often coupled with the assertion that White people are being treated unjustly and are suffering as a result. The idea that people of color are undeserving of success is often embedded in this sentiment.

*Then he said that Black on White crime is also very prevalent and that we should stop killing them because of their race, and that I have Black privilege. At that point, I was like… I didn’t want to know what he meant by that. If there is Black privilege, I haven’t seen it. I would like some. – Male respondent*

*“Why are you still calling yourself African American? You’re no more African than I am Australian!” and I was like, it’s not my fault that I was cut off from my heritage. I did not choose that life, but instances like that are where it gets sensitive. – Female respondent*

*I see a lot of people have problems with Porter Scholarship. That was something that was brought up in our class and one girl was like “I don’t understand why African Americans can have lower grades than me, then why do they get a scholarship?” – Female respondent*

### Pathologizing minority culture or appearance

This occurs when people of color are criticized due to real or perceived cultural differences in appearance, traditions, behaviors, or preferences.

*I went to a predominantly White school and lived in a predominantly White town in western Kentucky and one of my really close friends told me, “You would be the perfect girlfriend if you were White.” – Female respondent*

*The manager brought in this Black guy to interview. He was clean cut, he had dreads but they were nice and stuff. Like no big deal, he has dreads. And I know our doctor was like, “Is she going to hire him?” I was listening and I was like “Is this a racial thing?” Cause he looked perfectly fine: clean-cut, nice suit, super nice guy. – Female respondent*

Also embedded in this sentiment is the idea that Whiteness is preferred, and consequently there is something negative or shameful about a non-White identity. Hence, microaggressions may include statements that advance pronouncements of apparent Whiteness as complimentary. Our focus group participants overwhelmingly reported statements such as these to be upsetting and insulting.

*I was studying with this kid in my sociology class. And we had been studying for a while throughout the quarter and pretty much this time we were studying and he was like, “You know C., you’re not* *that* *Black. You’re pretty White.” – Male respondent*

### Second class citizen / ignored & invisible

This microaggression captures situations in which people of color are treated with less respect, consideration, or care than is normally expected or customary. This category is meant to include both the experience of being treated as a “second class citizen” (e.g., the preferential treatment of White individuals; [41, 42]), and the experience of being ignored, unseen, or invisible.

*My name is [redacted] and people think it’s difficult for people to say, and some people assume that it’s ghetto even though it is a last name. Whenever she [her former manager] would see me or ask me to do something she would be like, “Oh, I can’t think of your name.” It was going on for weeks to the point that it was getting ridiculous. I think that she felt like that because my name was harder or different that she didn’t need to try and learn it. She didn’t have enough respect for me to learn my name and treat me like everybody else. - Female respondent*

*With all the shootings that have been happening in the Black community, I kind of felt a certain way when I didn’t hear anything from my school that there was some kind of support for us. To just acknowledge that there are people that are here that can be affected, but with the Orlando shootings there was a different response. There were emails, there were ceremonies, and I was just like I thought they weren’t allowed to interfere…the first thing I said to myself was like, “They’re not allowed to probably bring it politics and other things into schools. That’s why they didn’t send an email.” But then there was such an overwhelmingly, overwhelming response to the Orlando shootings, I was like, “That’s not the case.” – Female respondent*

### Connecting via stereotypes

Many focus group participants described awkward situations in which White students attempted to communicate or connect through use of stereotyped speech or behavior, believing that will help them be accepted or understood.

*He just came up to me, and he was like so “Wassup?” And he’s like talking with his hands and doing all these [gestures]. Just like wassup, like trying to talk to me but using like things that he thinks like – I guess to connect with me – like cause we’re… I don’t know what it was, but it was just weird and made me feel uncomfortable. Um, so I just asked him. I was like, “What, like, what are you trying to say? What are you doing?” And basically I just had to end the conversation. … Why try to use like this hip cool language to try to connect when we could have had like a conversation just as well. – Female respondent*

This category can include using racial jokes or even racist epitaphs to try to fit in or as terms of endearment.

*She’s Caucasian, and like we hang around a lot of Black people. So she just generally has a pretty Black group of friends, you know, and she’s always dated African American people. And so she feels like it’s ok for her to freely use the N-word. – Female respondent*

### Exoticization and eroticization

This occurs when a person of color is treated according to sexualized stereotypes, or perceived differences are characterized as exotic in some way. Some examples shared by our participants included the following.

*I dated quite a few White women, and I agree that they fetishize us. They don’t really look at me as like a man. It’s ah “Oh, a Black man!” or a stereotypical big dude kind of thing. – Male respondent*

*I’ve actually been to a few frat parties, and I stopped going because every time I go they’ll be like, “Hey, the Black girl’s here!” They’ll be like, “Hey, can you twerk on me or something?” And I always get that, and I’m just like, ugh. And it’s really sad, because like White women will come up to me and ask, “Can you teach me how to twerk?” – Female respondent*

Many participants also shared microaggressions they had experienced surrounding their hair, and the frustration they felt over people asking pointed questions or attempting to touch it.

*When I don’t wear my headscarf, I have really curly thick hair. So it’s like, “Ohhh, can I touch it?” No, you can’t touch my hair. Or, yeah, “How do you get your hair like that?” I’m like, “It’s water. Just water.” Or “Is that a wig?” No it’s not a wig, it’s my hair. And then it has taken me awhile to like accept my hair, and how you know curly it is, and it’s high maintenance. And to accept my curl pattern and then to have people tell me, “Oh maybe you should wear your hair straight.” It’s like… it’s like a slap in the face. – Female respondent*

These types of microaggressions were not described in Sue et al.’s [41] original taxonomy, but are similarly represented in a category called “Sexual Objectification” in Sue and Spanierman [42].

### Avoidance and distancing

This occurs when people of color are avoided, or measures are taken to prevent physical contact or close proximity. This includes the exclusion of members of targeted groups through physical distancing. It can also include avoiding close relationships and difficult discussions about race.

*We were alternating group leaders to lead discussions about a paper we read for the week. And it was kind of like this random thing, so I was excited when it was my turn to be the group leader because I was interested in the subject. I had spent hours thinking of, you know, thoughtful questions to talk about, and then nobody showed up to my group… There was like five different group leaders, and so everyone kind of dispersed to the other four groups and no one showed up to my group, and I was just in tears because this has happened my whole life. Like no one has ever wanted to hear what I had to say. – Female respondent*

*I ride the bus every day, and so often like I’ll have like two open seats next to me, and like so much of a person avoids. And they’ll go choose to sit next to someone really close than have, like, have open space next to them. That happens to me a lot. – Male respondent*

### Environmental exclusion

Certain microaggressions that are more apparent on systemic and environmental levels have been defined previously as “environmental microaggressions” [41]. Environmental exclusion is a microaggression that occurs when someone’s racial identity is minimized or made insignificant through the exclusion of decorations, depictions, or literature that represents their racial group. It can also be used to describe situations where representations of people of color are not present in the classroom or workplace [25].

*I feel like there isn’t enough representation of prominent Black people anywhere, or people of color. Or see it at all. The news or on the Internet. I mean you can search for it but it’s not going to be like anywhere you can find. – Male respondent*

*We’re learning about what happens to White people when they get sick for instance. So, a White person is pale when they get anemia. Well, how do you tell if a Black person is anemic? I mean there is a way to tell, but they don’t ever talk about that. So I think that it is mostly geared towards White people, treating White people and not people of color. – Female respondent*

Diversity in leadership can be incredibly valuable to students’ interest and engagement. One noted:

*I finally have like a Black female professor that, like, I didn’t even realize it until I had her this semester, that I couldn’t really relate and get interested in the topics that I’m studying. I’m interested in them, obviously, but like I couldn’t get interested in them like I am now because she… I relate to her more. And so like when I first walked into my classroom at the beginning of the semester and saw a Black woman like [intakes full excited breath] I was like overwhelmed. I was excited. – Female respondent*

### Environmental attacks

The category of Environmental Attacks is intended to describe situations in which decorations or depictions pose a known affront or insult to a person’s cultural group, history, or heritage (e.g., buildings named after slave owners, Confederate monuments, Columbus Day). For example, on the topic of Confederate flags, participants reported feeling afraid and uncomfortable:

*Like oh my gosh, that’s so uncomfortable. I’m like “uh oh” … It’s like I’m unwanted in that area. You’re just like, oh my gosh, what if they do something to me? They must hate me, they don’t want me to be here. Like maybe, I should leave. – Female respondent*

*If you were to see a swastika or any other symbol of somebody who went through a similar situation they would immediately take it down, but anything that has to do with pertaining to the Black struggle, what we went through, they don’t really seem to acknowledge it. Just like they arrested that lady, I think it was in South Carolina, when she went up and took that flag down and she got arrested for it. That really makes me mad.. – Female respondent*

At one point, members of the executive office at one of the universities had dressed up in stereotypical Mexican garb for a Halloween party. Students expressed feelings of hurt about the event, especially because the university president had participated [50].

*Personally, I felt that although I’m not Hispanic, when he wore the sombrero and threw the party I felt it affected me to, because if you can disrespect those students at [this university] then you are disrespecting me as well. – Female respondent*

## Discussion

Overall, the students in this study reported a variety of microaggressive experiences on campus, and these caused distress, confusion, and led them to question their perceptions of events. Students were not quick to ascribe racist intentions to perpetrators, but often did so after careful evaluation of the situation and many times opted to ascribe no motivations to offenders at all. This is consistent with Essed [11], who explains that accounts reflect interpretations of reality-based inferences from the target’s general knowledge, and rational comparisons between racist and non-racist situations. The pain, frustration, and helplessness which racism often causes are strong incentives to carefully examine an event before judging it discriminatory, and lends further support to the need for as comprehensive a taxonomy as possible.

The accounts shared by our participants are not unlike accounts from other Black university students. In one sample of Black students attending a PWI Midwestern campus, students described their uncertainty and withdrawal from the heavy pressure to speak for a homogenous Black experience in light of both faculty and classmate pressure to do so [45]. In qualitative studies across the United States, including Ivy League universities, Black students report a variety of experiences that saturate both campus and social environments adjacent to schools that emphasize a lack of belonging, and Black men describe specific Black misandry that served as a constant sources of environmental stress [38, 39]. Young Black women’s experiences emphasize the challenging, narrow range of expectations and stereotypes, ranging from exotic sexualization to an expectation of strength or irrational anger [24]. These findings highlight the taxing necessity for Black college students to carefully monitor their environments for signs of threat, while simultaneously restricting and monitoring their own behavior to avoid the pitfalls of fulfilling a stereotype that leads to further racist mistreatment.

Subtle forms of racism, such as microaggressions, can be difficult to identify, quantify, and rectify because of their nebulous and unnamed nature. Although racial maltreatment exists on a continuum of discriminatory action ranging from gross and intentional to tiny unconscious slights [12], there is a need for a unified language in the study of the experience of this form of covert racism. The burgeoning research on the topic of microaggressions, while important in identifying the vast scope and depth of the problem, has made it increasingly difficult to identify such a common language that integrates multiple perspectives and provides an opportunity to adequately capture emerging categories. Our study identified 15 common categories of microaggressions as experienced by people of color, including the 9 originally described by Sue et al. [41].

In comparing our findings to Sue and colleagues’ 9 categories, which are also a part of the Sue and Spanierman [42] taxonomy, we find many similarities, and some notable differences as well. Sue et al’s “Alien in Own Land” was split into two distinct categories, “Not a True Citizen” and “Racial Categorization & Sameness” to differentiate questions about nationality to those surrounding ethnicity and race. Our category, “Assumptions About Intelligence, Competence, or Status” directly maps onto Sue et al.’s “Ascription of Intelligence,” with the addition of ascription of social class. Our category “False Colorblindness / Invalidating Racial or Ethnic Identity,” maps onto Sue et al.’s “Color blindness,” with the specification that colorblindness is only negative when it serves to invalidate an important facet of a person’s identity. Our category “Criminality or Dangerousness” directly maps onto Sue et al.’s “Criminality/assumption of criminal status” with no difference, and “Denial of Individual Racism” is identical to Sue et al.’s category of the same name, and likewise “Myth of Meritocracy,” which we clarify by adding “Race is Irrelevant for Success.”

We added a category called “Reverse Racism Hostility,” which in some ways extends “Myth of Meritocracy.” This category is represented in the literature by Lewis et al. [23] conceptualization of “White Resentment and Hostility about Affirmative Action” toward people of color and Clark et al.’s [3] theme of “Withstanding Jealous Accusations” in relation to indigenous people in Canada.

Our category called “Pathologizing Minority Culture or Appearance” extends Sue et al.’s category called “Pathologizing cultural values/communication styles,” by adding judgements about appearance. Our category called “Second Class Citizen / Ignored & Invisible” is the same as Sue et al.’s “Second-class citizen” but we added invisibility to the name to underscore that being unseen is also prevalent among people of color, especially Black women (e.g., [24]). Other new categories were “Connecting via Stereotypes” which has been noted in the literature in various contexts (e.g., [13]), and “Exoticization and Eroticization” which has come up repeatedly in both the quantitative and qualitative literature (e.g., [22, 29]). As noted previously, Sue and Spanierman [42] added a category called Sexual Objectification, but this only refers to women, whereas men of color are often sexually objectified as well.

We also added “Avoidance and Distancing” which is captured only a little by Sue et al.’s “Criminality/assumption of criminal status,” but this category seemed inadequate because there were many reasons apart from danger that people of color may be avoided [14]. We split Sue et al.’s “Environmental microaggressions” into two categories, the first focusing on macro-level exclusion and the second called “Environmental Attacks.” We split this from the larger category of environmental microaggressions to capture these particularly hurtful and often frightening depictions [7, 27], which have been an ongoing source of consternation, public attention, and institutional resistance [5, 50].

Finally, although we found evidence for a “tokenism” category, we did not have enough responses to formalize this. Tokenism is often described as the inclusion of individuals only because of their race for the illusion of inclusivity [25]. While this theme has been documented in the literature surrounding racial aggression, it was not specifically addressed by the moderators during the focus groups, thus the data did not yield exemplary material that would fall into this category. It is also not discussed by Sue et al. [41] or Sue and Spanierman [42].

The IPA approach utilized in this study may differ from expectations of those familiar with IPA research. Particularly, the final number of themes (15) is greater than the general approach to the identification of meta-themes most common in qualitative work. This is an artifact of the researchers’ goals and philosophy in approaching the topic. That is, this is not only an elaboration of themes revealed in the discussions of the focus groups, it is also an attempt to consider themes at a common level of analysis with existing microaggressions literature. This is reflected in the greater frequency of specific microaggression types in empirical research compared to the less frequent utilization of proposed meta-categories of microaggressions (i.e., microassault, microinsult, and microinvalidation; [41]).

### Limitations and future directions

This study is not without limitations. The sample included self-identified predominately Black students from three institutions in only two geographical regions. Some types of microaggressions are more common for Black people than people in other ethnoracial groups [42]. Future work on this topic may result in an updated taxonomy that accounts for the increasing intersectionality of marginalized identities such as gender, sexual orientation, or religion. For example, Donovan et al. [9] assessed the intersectionality of race and gender among female graduate and post-graduate students, and Weber et al. [46] interviewed graduate students that identified as sexual minorities. These studies explicated unique categories not represented here but relevant for those specific groups.

This paper has focused on a classification system based the actions of perpetrators, but there may be better and more equitable ways to classify these behaviors. For example, in some situations it may be better to classify microaggressions based on the intention of the perpetrator (e.g., superordinate forms of microaggressions; [15, 41]) or the impact on the victim (e.g., [3]). Likewise, future research should examine the differential harms to victims resulting from the different types of microaggressions described herein.

## Conclusions

Without an adequate understanding of the illusive dynamics of subtle racism, microaggressions will remain invisible and harmful to the well-being, self-esteem, and standard of living of people of color. While previous literature has either embraced the taxonomy developed by Sue et al. [41], or proposed a novel taxonomy unique to specific data, this study utilizes the Sue et al. [41] and Sue and Spanierman’s [42] framework as a starting point toward understanding our own focus group findings. We also move that work forward by splitting the original “Alien in One’s Own Land” and “Environmental Microaggressions” into two categories, and drawing attention to the need to further examine microaggressions that typify connecting though stereotypes and tokenism.

Although this paper is not the last word on how to best categorize microaggressions, we hope this serves as a step in the right direction and call for more work in this area based on a systematic review of research of studies to date. Ultimately, a unified language of microaggressions may better allow for improved measurement of this construct in both qualitative and quantitative studies. It may also facilitate self-report of microaggressions by aggressors to better enable them to honestly and earnestly explore personal biases and minimize the associated negative social outcomes. It may further relieve the onus of those who are the recipients of repeated microaggressions to “prove” the validity of their perceptions and experiences [1].

It is our hope that this work will contribute to moving the field toward a shared language of microaggressions, and thus a consensus will emerge across multiple fields of interest surrounding the study of prejudice and racism.

## Data Availability

Data is available from the corresponding author (MTW) by reasonable request.

## Abbreviations

GPA: Grade point average
HPA: Hypothalamic-pituitary-adrenal
IPA: Interpretative phenomenological analysis
IRB: Institutional Review Board
PWI: Predominately White Institutions
